# Dual-Emission FRET-PCR Outperforms SYBR Green and EvaGreen for Accurate Discrimination of Primary Canine Dermatophytes: *Microsporum canis*, *Nannizzia gypsea*, and *Trichophyton mentagrophytes*

**DOI:** 10.3390/jof11100708

**Published:** 2025-09-30

**Authors:** Nneka Vivian Iduu, Rae Kantzler, Donna Raiford, Brenda Bixler, Kelly Chenoweth, Chengming Wang

**Affiliations:** 1 Molecular Diagnostics Laboratory, College of Veterinary Medicine, Auburn University, Auburn, AL 36849, USA; nvi0001@auburn.edu (N.V.I.); ark0066@auburn.edu (R.K.); kjc0063@auburn.edu (K.C.); 2 Department of Pathobiology, College of Veterinary Medicine, Auburn University, Auburn, AL 36849, USA; drr0004@auburn.edu (D.R.); bixlebm@auburn.edu (B.B.); 3 Bacteriology and Mycology Laboratory, College of Veterinary Medicine, Auburn University, Auburn, AL 36849, USA

**Keywords:** dermatophytes, *Microsporum canis*, *Nannizzia gypsea*, *Trichophyton mentagrophytes*, FRET-qPCR

## Abstract

Conventional diagnosis of dermatophytosis relies on fungal culture and microscopic examination, methods that are often time-consuming and lack sensitivity. This study aimed to develop and compare real-time PCR assays for the simultaneous detection and differentiation of three major dermatophytes in dogs: *Microsporum canis*, *Nannizzia gypsea*, and *Trichophyton mentagrophytes*. Three qPCR platforms targeting the chitin synthase 1 (*CHS1*) gene—SYBR Green, EvaGreen, and dual-emission fluorescence resonance energy transfer (FRET)—were evaluated. The FRET assay demonstrated the highest performance, achieving a detection limit of a single gene copy per reaction and producing distinct melting profiles that enabled accurate species discrimination (*M. canis* ~56.1 °C, *N. gypsea* ~53.0 °C, *T. mentagrophytes* ~51.8 °C). In contrast, SYBR Green and EvaGreen assays showed reduced sensitivity and cross-reactivity with non-target fungi. All assays were validated using three ATCC reference strains, ten clinical isolates of the target dermatophytes, and nine additional fungal species, including *Nocardia*, *Aspergillus*, *Fusarium*, *Sporothrix*, and *Candida*. Overall, FRET-qPCR exhibited a 100% specificity and a detection limit of one copy of target gene per reaction, offering a rapid, reliable tool for accurate diagnosis and molecular surveillance of dermatophytosis in companion animals.

## 1. Introduction

*Microsporum canis*, *Nannizzia gypsea*, and *Trichophyton mentagrophytes* are the most common dermatophytes associated with superficial fungal infections in canines [1,2]. These keratinophilic fungi invade the skin, hair, and claws, causing dermatophytosis, a contagious condition of veterinary and public health importance due to its zoonotic potential [3].

Accurate identification and differentiation of these infecting species are essential for guiding treatment, limiting transmission, and supporting epidemiological surveillance [4]. This is especially important given their distinct transmission routes [5]. *M. canis* is primarily zoophilic and highly transmissible through direct animal contact, while *N. gypsea* is geophilic, typically acquired from contaminated soil, and *T. mentagrophytes* includes both zoophilic and anthropophilic variants, with recognized zoonotic transmission [5,6,7,8].

Traditionally, diagnosis of dermatophytosis relies on clinical history, physical examination, direct microscopic examination with potassium hydroxide (KOH), and fungal culture [2,9]. Although mycological culture remains the gold-standard test, it requires prolonged incubation periods (2–4 weeks) [10] and may yield inconclusive results due to phenotypic overlap among species or contamination by environmental fungi [11,12]. Consequently, conventional methods are often time-consuming, may lack sensitivity or specificity, and can delay clinical decision-making [10,12].

Molecular diagnostic methods, particularly real-time PCR, offer technical advantages in speed, sensitivity, and specificity over conventional methods [2,13,14,15]. However, assay development for dermatophytes is challenging due to the need for both broad detection and precise species differentiation, along with consistent assay performance. Melting-temperature-based approaches employ different strategies for species identification. DNA-binding fluorescent dyes, such as SYBR Green and EvaGreen, are widely used in molecular assays. SYBR Green is a well-established intercalating dye for monitoring double-stranded DNA amplification [16], while EvaGreen—a newer-generation dye—offers improved high-resolution melting properties and reduced inhibitory effects [16,17]. Alternatively, fluorescence resonance energy transfer (FRET)-based real-time qPCR integrates the sensitivity of qPCR with the discriminatory capacity of melting curve analysis, enabling both detection and precise identification of target organisms via characteristic melting temperature profiles [18,19].

This study aimed to develop a rapid, highly sensitive, and specific qPCR assay for the simultaneous detection and differentiation of *M. canis*, *N. gypsea*, and *T. mentagrophytes.* By providing an efficient molecular tool for pathogen identification, this assay has the potential to improve diagnostic accuracy, facilitate timely clinical decisions, and support effective control of dermatophytosis in companion animals.

## 2. Materials and Methods

### 2.1. Fungal Strains

Three ATCC reference strains, including *Microsporum canis* (ATCC 36299), *Nannizzia gypsea* (formerly *Microsporum gypseum*, ATCC 10215), and *Trichophyton mentagrophytes* (ATCC 18748), were purchased for assay development. In addition, the ten fungal isolates (four *Microsporum canis*, three *Nannizzia gypsea*, and three *Trichophyton mentagrophytes)* used in this study were obtained from the Bacteriology and Mycology Diagnostic Laboratory at the College of Veterinary Medicine, Auburn University. In addition, to evaluate assay specificity, non-dermatophyte isolates were used as negative controls, including *Aspergillus niger*, *A. flavus*, *A. fumigatus*, *Candida albicans* (isolates 3225-6-97 and ATCC 90328), *Candida laurentii* (CDC 18802), *Candida parapsilosis* (ATCC 22019), *Nocardia asteroides*, *Fusarium* (strain 883-2-16), and *Sporothrix schenckii*.

### 2.2. Genomic DNA Extraction

Fungal genomic DNA was extracted using a combination of mechanical disruption and automated magnetic-bead-based purification. Four 3.0 mm ceramic sterile beads were added to each of the fungal samples previously placed in stabilization buffer, and these were homogenized in a shaker (Bertin Technologies, Montigny-le-Bretonneux, France) for four cycles of 30 s at 7000 rpm with a 120 s break interval between each cycle. Following homogenization, samples were centrifuged to remove cellular debris. The supernatant was processed using the IndiMag 2 automated magnetic-bead-based nucleic acid isolation system (INDICAL Inc., Orlando, FL, USA) with prefilled reagent cartridges, according to the manufacturer’s instructions. Genomic DNA was eluted in 100 μL of elution buffer and stored at −20 °C until further analysis.

### 2.3. Primer and Probe Design

Available representative genome sequences of *Microsporum canis* (FJ897700, PQ588604, PQ588606, PQ588609, XM002843156), *Nannizzia gypsea* (MK752533, KU705508, MK752537, MK752539, MK752541), and *Trichophyton mentagrophytes* (FJ897704, KM355549, MT273261, MT273262), as well as other fungi (*Arthroderma otae* AB003563; *Arthroderma persicoir* AB006983; *Arthroderma quadrifidum* AB050584; *Arthroderma simii* AB003564; *M. incurvatum* AB003562, MK752543; *T. benhamiae* XM003011160; *T. rubrum* AB018564; *T. terrestre* FJ897706; *T. verrucosum* MT373258, EU363514, MT273256; *T. interdigitale* AB003565), were retrieved from GenBank and aligned using the Vector NTI software v11.5 (Invitrogen, Carlsbad, CA, USA). Primers were designed to target conserved regions of the chitin synthase 1 (*CHS1*) gene, ensuring specificity for each dermatophyte species while minimizing cross-reactivity with other dermatophyte and non-dermatophyte fungi.

The primer set was evaluated using three real-time PCR detection formats: SYBR Green, EvaGreen, and a hydrolysis-probe-based FRET system. All primer and probe sequences were synthesized by Integrated DNA Technologies (Coralville, IA, USA) and targeted a 200 bp amplicon of CHS1. The sequences were as follows: forward primer: 5′-TTCGCCCGMACMATGGC-3′; reverse primer: 5′-ACCTRCTGTTTGGCAATGCC-3′. For the FRET assay, the following probes were used: anchor probe: 5′-TGCTCRCGCACCAGCAGCAA-6-FAM-3′; reporter probe: 5′-Cy5-LCR640-ACATGGGGCAAGGAAGCCTGG-Phos-3′. The anchor probe was labeled with 6-FAM and excited at 488 nm, while the reporter probe was labeled with LC Red 640 and emitted fluorescence at ~640 nm upon FRET excitation.

Sequence alignment showed 100% conservation of primer and anchor probe regions across all three dermatophytes, with species-specific polymorphisms in the reporter probe region: *N. gypsea* matched the probe sequence exactly, *M. canis* contained two mismatches at the probe ends, and *T. mentagrophytes* contained two central mismatches. These variations were designed to generate distinct melting temperatures for species differentiation. SYBR Green and EvaGreen assays were performed using the same primer pair without probes.

Thermal cycling for differential dermatophyte qPCR was performed using a LightCycler^®^ 480 II real-time PCR platform (Roche Diagnostics, Indianapolis, IN, USA). For the FRET assay, cycling conditions were optimized based on protocols described by Iduu et al. [19] and Gong et al. [18], involving 18 high-stringency step-down cycles followed by 30 relaxed-stringency fluorescence acquisition cycles, with an annealing temperature of 57 °C. For SYBR Green and EvaGreen assays, the high-stringency phase comprised 6 cycles of 10 s at 95 °C, 10 s at 70 °C, and 10 s at 72 °C; 9 cycles of 10 s at 95 °C, 10 s at 68 °C, and 10 s at 72 °C; and 3 cycles of 10 s at 95 °C, 10 s at 66 °C, and 10 s at 72 °C. This was followed by 30 relaxed-stringency fluorescence acquisition cycles for SYBR Green and 40 cycles for EvaGreen [20] of 10 s at 95 °C, 10 s at 57 °C, and 10 s at 72 °C.

The melting curve analysis was determined by monitoring the fluorescence from 45 °C to 80 °C, and fluorescence data were continuously collected using the F4/F1 emission ratio. The melting temperature (*T*_m_) was determined by plotting the negative derivative of fluorescence with respect to temperature (−d(F4/F1)/dT), yielding distinct melting peaks corresponding to specific amplicon dissociation.

The sensitivity of the *Dermatophyte* qPCR was verified using the gBlock gene fragments containing the conserved CHS1 region of *Nannizzia gypsea*, *Microsporum canis*, and *Trichophyton mentagrophytes*, which were synthesized by Integrated DNA Technologies (Coralville, IA, USA). Based on the molecular weight of each gBlock DNA fragment, 10-fold serial dilutions ranging from 10^4^ to 10^0^ copies per 10 µL reaction were prepared in triplicate to determine the assay’s detection limit. The specificity of the qPCR was verified using the genomic DNA of the ten clinical non-dermatophyte isolates. The PCR products were sent to ELIM Biopharmaceuticals (Hayward, CA, USA) for Bidirectional Sanger sequencing.

Finally, gBlock fragments, genomic DNA from all ATCC strains, and clinical dermatophyte and fungal isolates were tested using FRET, SYBR Green, and EvaGreen qPCR formats. Performance was compared in terms of sensitivity, specificity, and ability to differentiate *M. canis*, *N. gypsea*, and *T. mentagrophytes*. All PCR products were sent to ELIM Biopharmaceuticals (Hayward, CA, USA) for Bidirectional Sanger sequencing.

## 3. Results

The three qPCR detection formats—SYBR Green, EvaGreen, and FRET—exhibited distinct amplification behaviors and melting curve profiles, enabling differentiation of *Microsporum canis*, *Nannizzia gypsea,* and *Trichophyton mentagrophytes*. Each platform showed characteristic performance patterns across both target and non-target species.

**Figure 1:**
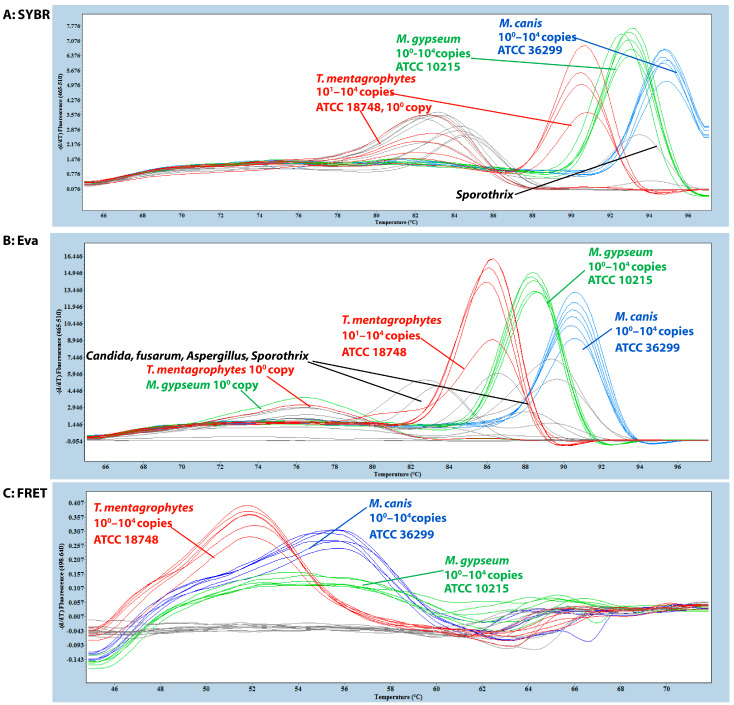
Melting-temperature-based differentiation of three dermatophyte species in SYBR Green, EvaGreen, and FRET-based qPCRs. All assays targeted the *CHS1* gene region, with melting temperature (*T*_m_) determined by HRM analysis from 45 °C to 80 °C. Testing included synthetic gBlocks (10^0^–10^4^ copies per reaction), ATCC reference strains, clinical isolates, and non-dermatophyte controls (*Nocardia asteroides*, *Aspergillus* spp., *Fusarium*, *Sporothrix schenckii*, and *Candida* spp.). (**A**) SYBR-Green-based qPCR: *Microsporum canis* (blue) and *Nannizzia gypsea* (green) showed consistent detection across all dilutions (10^0^–10^4^ copies). *Trichophyton mentagrophytes* (red) exhibited inconsistent detection and variable Tm values in 10^0^ copies and ATCC strains. Non-specific melting peaks were observed in most non-dermatophyte controls (grey). (**B**) EvaGreen-based qPCR: Detection sensitivity varied by species: *M. canis* (blue) detected from 10^0^–10^4^ copies, while *N. gypsea* (green) and *T. mentagrophytes* (red) were reliably detected only from 10^4^–10^1^ copies. All ATCC strains were consistently detected; however, *T. mentagrophytes* showed reduced peak intensity. Non-specific peaks occurred in non-dermatophyte controls (grey). (**C**) FRET-based qPCR: All three species showed distinct, consistent Tm values: *T. mentagrophytes* (red, ~51.8 °C), *M. canis* (blue, ~56.1 °C), and *N. gypsea* (green, ~53.0 °C). Complete detection across all dilutions (10^4^–10^0^ copies) with ATCC strains matching synthetic gBlock profiles. No cross-reactivity detected in non-dermatophyte controls (grey).

SYBR-Green-based qPCR detected all three dermatophyte species using synthetic gBlock fragments (10^4^–10^0^ copies) containing the *CHS1* target region but failed to correctly amplify target DNA at low template concentrations (10^0^ copies) for *T. mentagrophytes.* ATCC reference strains were correctly identified and differentiated for *M. canis* and *N. gypsea*; however, melting peaks for *T. mentagrophytes* varied compared to those of the synthetic gBlocks (Figure 1A). A similar differentiation pattern to that observed in the ATCC reference strains was also seen in the clinical isolates. Additionally, non-specific melting peaks were detected in most non-dermatophyte samples, including *Nocardia asteroides*, *Aspergillus niger*, *A. flavus*, *A. fumigatus*, *Fusarium*, *Sporothrix schenckii*, *Candida albicans*, *Candida laurentii*, and *Candida parapsilosis.* (Figure 1A).

EvaGreen-based qPCR detected all three dermatophyte species from the synthetic gBlocks, including all ATCC reference strains, although the *T. mentagrophytes* strain exhibited a lower melting peak. Similar to SYBR Green, EvaGreen failed to correctly detect low-copy targets (10^0^ copies) for species such as *T. mentagrophytes* and *N. gypsea*. Non-specific melting peaks were also observed in non-dermatophyte samples (Figure 1B). In clinical isolates, detection patterns resembled those of the ATCC strains, with variation in *T. mentagrophytes* melting peaks.

The FRET-based qPCR successfully detected and differentiated the three dermatophyte species using synthetic gBlock fragments. It consistently amplified targets across 10^0^ to 10^4^ copies per reaction for all species, demonstrating superior analytical sensitivity. Distinct melting peaks were observed for each species: *T. mentagrophytes* (~51.8 °C), *M. canis* (~56.1 °C), and *N. gypsea* (~53.0 °C), with *N. gypsea* showing a broader and lower peak (Figure 1C). No fluorescence signal was detected in any of the non-dermatophyte controls. The FRET-qPCR correctly identified all ATCC reference strains and ten clinical isolates, with melting profiles matching those of the respective reference strains (Figure 1C). DNA sequencing validated these identifications.

## 4. Discussion

The FRET-based qPCR assay developed in this study offers a sensitive, specific, and rapid method for species-level identification of the three most prevalent dermatophytes in canines: *Microsporum canis*, *Nannizzia gypsea*, and *Trichophyton mentagrophytes*. Unlike conventional fungal culture and microscopy, which may take 2–4 weeks and suffers from low sensitivity, especially in subclinical or pretreated cases, this molecular method produces results within 2.5 h, reducing turnaround time and contamination risk [10,21].

Comparative evaluation revealed notable limitations in dye-based qPCR systems. Although SYBR Green offers ease of implementation [22], it produced non-specific fluorescence signals and exhibited reduced sensitivity at low DNA concentrations. EvaGreen, while offering superior melting curve resolution, did not overcome these limitations. In contrast, the FRET assay’s dual-probe design combines sequence-specific fluorescence resonance energy transfer with high-resolution melting curve analysis, resulting in superior specificity and reliable differentiation. This capability is particularly valuable for clinical specimens that may contain mixed fungal populations or environmental contaminants.

The relatively slow growth rates of dermatophytes often complicate timely and sufficient DNA extraction. Low DNA concentrations can hinder accurate detection and species-level identification [23,24]. Dye-based systems are susceptible to non-specific amplification from primer–dimer formation or background fluorescence, complicating melt peak interpretation [25]. Even TaqMan-based qPCR assays, which utilize hydrolysis probe detection, have occasionally produced false-negative results, particularly under conditions of limited DNA yield or sample degradation [14]. Such issues were not encountered with our FRET platform, which consistently yielded specific, reproducible melt curves across all tested concentrations, including single-copy templates.

Dye-based melting temperatures depend on the GC content and thermal stability of the entire amplicon, which can introduce variability. In contrast, FRET-qPCR melting temperatures are defined solely by the probe-binding region, offering greater control and reproducibility. In this assay, primers and the anchor probe were fully conserved among the three dermatophytes, while the reporter probe targeted a highly conserved region containing species-specific mismatches. This design consistently produced distinct melting temperatures, giving the FRET platform a clear advantage over SYBR Green and EvaGreen systems.

The use of a short (~200 bp) amplicon likely contributed to the assay’s high amplification efficiency and robust melting behavior, offering a technical advantage over previously described systems [2]. Shorter amplicons are generally more tolerant of DNA degradation, less affected by PCR inhibitors, and better suited for clinical diagnostics, especially when working with low-abundance fungal DNA [26,27].

Clinically, *M. canis*, *N. gypsea*, and *T. mentagrophytes* are the main causative agents of dermatophytosis in canines [1,2,28]. Rapid species-level identification supports early initiation of targeted therapy and appropriate infection control measures. Species identification is critical because these dermatophytes differ in their zoonotic potential, treatment response, and environmental persistence [7,28].

The *T. mentagrophytes* complex presents particular diagnostic challenges, comprising pleomorphic fungi that exhibit both anamorphic (asexual) forms, including *T. mentagrophytes*, *T. interdigitale*, and *T. indotineae*, and teleomorphic (sexual) forms such as *Arthroderma benhamiae*, *Arthroderma simii*, and *Arthroderma vanbreuseghemii*. *Trichophyton indotineae*, a recently described species, is particularly notable for its resistance to terbinafine [29]. These variants show variable transmission routes, antifungal resistance patterns, and treatment responses [7]. While sequencing and nucleotide BLAST analysis of PCR amplicons confirmed assay accuracy, isolates identified as *T. mentagrophytes* in this study exhibited high sequence identity with *T. interdigitale* and *A. benhamiae*, reinforcing the taxonomic complexity within this species complex. This challenge has been widely recognized in the literature [30], and in epidemiological contexts, additional sequencing following PCR may be necessary for precise subspecies identification, suggesting that sequencing may be necessary in epidemiological investigations. One limitation of this study is that the primer and probe design did not include all sequences from human- and animal-pathogenic dermatophytes.

The *CHS1* gene, used in this study, encodes chitin synthase, an essential enzyme that builds chitin, the structural polysaccharide of the fungal cell wall. Because chitin is indispensable for fungal growth, CHS1 is a single-copy, conserved, housekeeping gene across dermatophytes [31]. However, the genes targeting the translation elongation factor 1-α gene (tef-1α) and β-tubulin (BT2/TUBB) could be used to better differentiate between the sexual and asexual forms of the *T. mentagrophytes* complex than the *CHS1* gene [32,33].

Traditionally, positive controls and quantitative standards were generated from cultured organisms. Although reliable, this approach was limited by biosafety concerns, variability in culture conditions, and restricted availability of certain pathogens. The use of synthetic plasmids and gBlocks encoding the target PCR amplicon regions overcomes these issues by providing a safe, stable, and precisely defined template. This not only enhances reproducibility and standardization across assays but also facilitates broader applicability in diagnostic development and validation without the need to handle live organisms.

FRET PCR, used in this study, provides high specificity and sensitivity but has several limitations. Probe design is complex, requiring precise optimization of length, melting temperature, and target-binding positions; poor design can reduce energy transfer, weaken signals, or cause cross-reactivity. Additionally, not all PCR instruments support FRET detection, and the dynamic range may be narrower than dye-based qPCR if probe hybridization is suboptimal. Improvements can be achieved through careful in silico probe design, selection of high-quality fluorophores with minimal photobleaching and spectral overlap, and, for multiplex assays, using probes with distinct emission spectra to minimize crosstalk and enhance reliability.

Although the current work validated the assay using synthetic gBlock fragments, ATCC reference strains, and clinical isolates, future testing should include direct clinical specimens such as hair and skin scrapings. Previous studies have demonstrated the feasibility of *CHS1*-targeted qPCR on hair and skin scrapings with appropriate sample processing [2]. The strong performance of our assay at low DNA concentrations suggests it could be highly effective in these contexts. Expanding validation to include geographically diverse isolates will further ensure assay robustness against strain variation. Future efforts will focus on applying whole-genome next-generation sequencing (NGS) for fungal diagnostics. This approach enables simultaneous detection of all pathogenic fungi in a sample, including rare or novel species, and provides genomic insights into virulence and antifungal resistance. NGS also supports high-throughput screening and more precise epidemiological analyses. Overall, it offers greater sensitivity, specificity, and flexibility compared to conventional PCR-based methods.

## 5. Conclusions

In conclusion, this FRET-based qPCR assay represents a significant advancement in identifying *M. canis*, *N. gypsea*, *and T. mentagrophytes*. It offers superior analytical performance over existing molecular approaches, overcomes key limitations of culture-based methods, and is well-suited for clinical implementation. Its rapid turnaround, high specificity, and tolerance for low DNA inputs make it particularly valuable for routine diagnostic use, with the potential to improve treatment outcomes and reduce transmission risk through timely and accurate species identification.

## Data Availability

The original contributions presented in this study are included in the article. Further inquiries can be directed to the corresponding author.
